# Estimation of anaerobic threshold and peak oxygen uptake from tracheal sound during cycle ergometer cardiopulmonary exercise test

**DOI:** 10.1186/s12938-026-01540-7

**Published:** 2026-02-27

**Authors:** Qi Zhang, Nasim Montazeri Ghahjaverestan, Cristina de Oliveira Francisco, Muammar Muhammad Kabir, Md. Saiful Hoque, Shahram Kharabian Masouleh, Paul Oh, Susan Marzolini, Azadeh Yadollahi

**Affiliations:** 1 KITE- Toronto Rehabilitation Institute, University Health Network, Toronto, ON Canada; 2 Faculty Electrical Engineering, University of Toronto, Toronto, ON Canada; 3 Institute of Biomedical Engineering, University of Toronto, Toronto, ON Canada; 4 Respirology, Faculty of Medicine, University of Toronto, Toronto, ON Canada; 5 Faculty of Kinesiology and Physical Education, University of Toronto, Toronto, ON Canada; 6 Electrical and Computer Engineering, Queen’s University, Kingston, ON Canada

**Keywords:** Cardiopulmonary exercise test, Acoustics, Tracheal sound, Anaerobic threshold, Oxygen uptake, Signal processing

## Abstract

Cardiopulmonary exercise test (CPET) is widely used for assessing exercise capacity of individuals through ventilatory anaerobic threshold (AT) and peak oxygen uptake (VO_2peak_). However, CPET requires specialized equipment, resources, time, and trained personnel. In this study, we investigated the feasibility of estimating ventilatory AT and submaximal VO_2peak_ using tracheal respiratory sounds recording with a wearable sensor (*The Patch*) during incremental cycle ergometer testing. Twenty-four healthy adults (18 females, age: 31.0 ± 9.1 years, body mass index: 23.5 ± 3.3 kg/m^2^) underwent CPET with simultaneous tracheal sounds recording. Acoustic features (sound energy, sound intensity, respiratory rate, and ventilation) were extracted. AT was identified from breakpoints in acoustic features and compared with reference AT from ventilatory gas analysis. VO_2peak_ was estimated using random forest regression models with subject-specific and subject-independent training models. Agreement with CPET was investigated using Bland–Altman analysis. Ventilatory AT was detected in 84% (sound energy) and 96% (sound intensity) of participants using *The Patch*. Workload stage agreement with CPET was 88% and 100% for sound energy and intensity, respectively, with AT timing within 20 s in 62% and 92% of cases. The subject specific model estimated VO_2peak_ with minimal bias [confidence intervals] of − 1.01 [− 3.04 to 1.02] (mL/kg/min), while the subject-independent validation model exhibited wider variability of − 1.28 [− 12.39 to 9.82] (mL/kg/min). Our results shows that tracheal sound analysis can estimate ventilatory AT and provides a promising approach for non-invasive exercise capacity assessment. On the other hand, the estimation of VO_2peak_ shows promise but requires further validation in maximal effort protocols.

**Supplementary Information:**

The online version contains supplementary material available at 10.1186/s12938-026-01540-7.

## Introduction

Cardiovascular, respiratory and related disorders are the major causes of 43% of deaths worldwide [1]. Patients with cardiovascular or respiratory disorders experience declines in physical performance and exercise tolerance [2, 3]. The quantitative assessment of exercise tolerance is exercise capacity, or the functional capacity of the human body to perform physical exercise at maximum capacity [4]. Poor exercise capacity is a modifiable risk factor for cardiorespiratory disorders [5], and a strong predictor of mortality for healthy individuals [6], and those with cardiovascular [6–8] and respiratory disorders [9]. The gold standard method for measuring exercise capacity is the cardiopulmonary exercise test (CPET). However, the CPET system is expensive, needs trained professionals to perform the test, and its application is limited within hospitals, research centers, and a handful of high-performance training centres [10]. Therefore, there is a need to develop low-cost alternative methods to assess exercise capacity.

CPET is an important tool to identify cardio-respiratory disorders and improve the effectiveness of physical exercise prescription [2, 11–13]. During CPET, incremental increases in exercise intensity are used to bring the individual to the limit of exercise tolerance [14]. Then, data from the heart rate (HR) monitor or electrocardiogram and gas exchange analyzer [2, 11, 15] are used to assess the indices of submaximal and maximal exercise capacity, including ventilatory anaerobic threshold (AT) and peak of oxygen uptake (VO_2max_) [13, 16, 17]. Ventilatory AT is a highly reproducible measure that indicates the oxygen uptake, where there is a transition from predominantly aerobic to anaerobic metabolism [13, 16]. The ventilatory AT and VO_2peak_ can be used to evaluate the risk of mortality and cardiovascular complications and to determine individualized exercise intensity prescriptions for rehabilitation and preventive exercise programs [18, 19].

While CPET measures both indices, ventilatory AT is typically achieved at submaximal effort and is a critical marker of functional endurance; this makes it a highly relevant clinical target, particularly for individuals unable to sustain maximal exertion, motivating our specific focus on this parameter. Several studies have explored different modalities to estimate measures of exercise capacity. One of the first methods to estimate ventilatory AT, was to detect the breakpoint in respiratory rate or find the amplitude of plateau in the variance of reciprocal tidal volume measured during exercise [20]. These studies were able to predict AT with more than 80% agreement with the reference values. However, measuring gas exchange is sensitive to leakage and body motion, and can be uncomfortable and change the pattern of breathing if it requires subjects to wear a mask or mouthpiece during exercise [21]. Another modality was heart rate variability, which has been used to estimate both AT [20] and VO_2peak_ [22, 23] during incremental exercise with a strong correlation *(r = 0.82–0.89)* and non-significant differences between the estimated indices and reference measures in the healthy population [22]. Moreover, HR [23] and submaximal VO_2_ [24, 25] have been used for estimating VO_2max_ with strong agreement between sex or age groups, whereas HR alone has a large standard error for estimating VO_2max_. In patient populations, models powered by artificial intelligence analyzed ECG signals and estimated the reference AT with the correlation of *r* = *0.87* with an average error of − 0.05 mL/kg/min [26]. A Hager correlation of *(r = 0.93)* was achieved for estimating instantaneous VO_2_ from analyzing ECG in combination with accelerometery [17, 27]. Finally, lactate threshold measured using near-infrared LED technology embedded in a compression calf sleeve was used to estimate AT with an error of less than *10%* [28]. One challenge associated with the modalities based on ECG or lactate measurement for estimating exercise indices is that they require acceptable contact to the skin, which can be compromised by body movements and sweating during exercise. In contrast, the suprasternal notch offers an anatomically stable recording site with minimal muscle contraction and skin deformation [29–31]. Furthermore, unlike electrical sensors, where signal quality is highly sensitive to sweat-induced impedance changes, acoustic sensors rely on mechanical coupling, which makes them potentially more resilient to perspiration during intense exercise.

In many studies, tracheal sounds have been used for assessing respiration [32, 33] and HR [33, 34], while the estimation of AT, and VO_2peak_ from tracheal sounds have remained underexplored. To address the gap in current methods to estimate exercise capacity measures and present a cost-effective and convenient modality for estimating exercise indices, we used acoustical respiratory signals that were recorded over the trachea. Previously, we developed algorithms to remove ambient noise during CPET and estimate respiratory rate from tracheal sounds with an error of < *1.2* breaths per minute [35]. In this proof-of-concept study, we investigated the hypothesis that tracheal sounds recorded during CPET can be used to estimate the exercise indices of AT and VO_2peak_.

## Results

Twenty-four participants (*6* male) with sedentary lifestyle (assessed by Baecke’s questionnaire), with age of *31* ± *9.1* years, BMI of *23.5* ± *3.3* kg/m [2], reference ventilatory AT (in time) of *381* ± *67* s and reference VO_2peak_ of *27.1* ± *6.7* mL/kg/min were included in the study.

### Ventilatory *AT estimation*

**Fig. 1:**
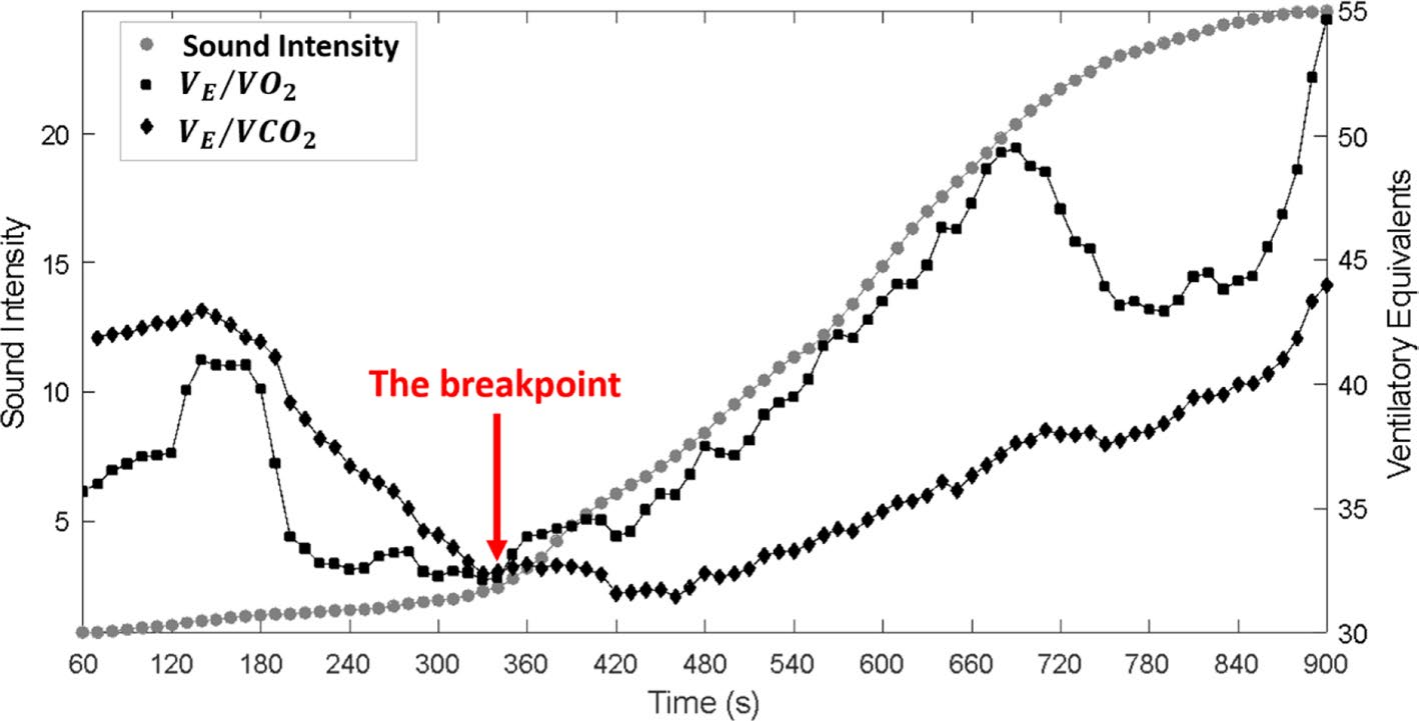
Comparison of the ventilatory anaerobic threshold determined from the sound intensity and ventilatory equivalents.The (Nadir of V_E_/VO_2_ occurred around 340 s, while V_E_/VCO_2_ was still decreasing. The breakpoint in sound intensity also occurred at 340 s, which agrees with the determination through CPET

Figure 1 illustrates the breakpoint of sound intensity and the related nadir of ventilatory equivalents (V_E_/VO_2_, V_E_/VCO_2_), which were used for reference ventilatory AT determination. Table 1 summarizes accuracy of different features to determine ventilatory AT. The proposed algorithms were able to detect AT in 20 participants (84%) based on sound energy and in 23 participants (96%) based on sound intensity. The time of AT was similar to those based on the reference values (time difference ≤ 20 s) using sound energy in 61.9% of the participants and using sound intensity in 91.7% of the participants. The AT workload determined by sound energy and sound intensity was the same as those based on the reference value in 88% and 100% of participants, respectively.

**Table 1:** Summary of the accuracy in ventilatory AT determination results

	Sound energy	Sound intensity
Successful detection	84%	96%
Accuracy of workload	88%	100%
Time difference < 20 s	61.90%	91.67%

**Fig. 2:**
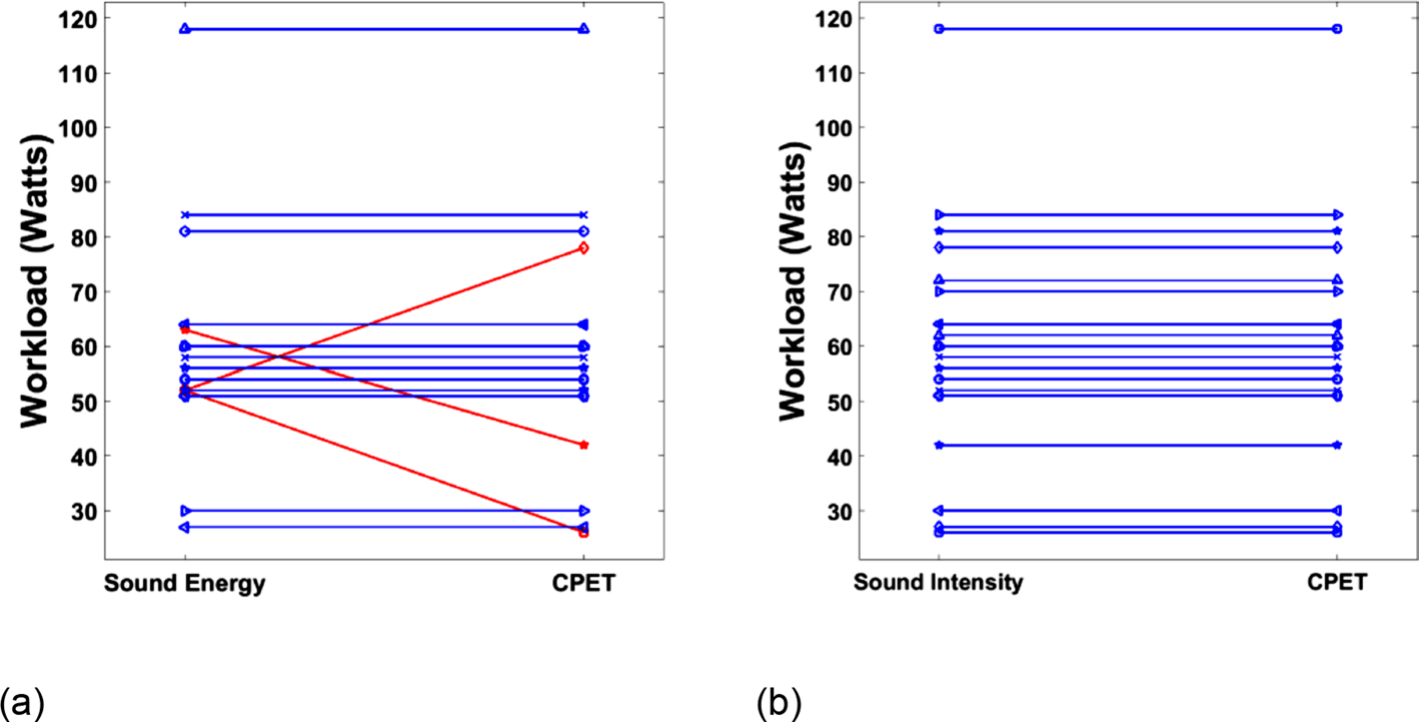
Workload (Watts) at the ventilatory AT determined by the CPET: **a** sound energy, **b** sound intensity

**Fig. 3:**
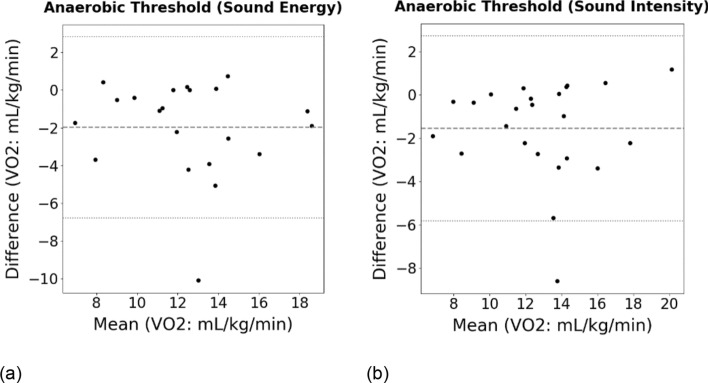
Bland–Altman analysis of the VO_2_ at ventilatory AT determined by the CPET: **a** sound energy, **b** sound intensity

Clinically, the *V̇*O_2_ at the timepoint of ventilatory AT is reported and the workload at the corresponding exercise stage is used for exercise prescription. Figure 2 shows the workload according to *The Patch* (Sound energy and Sound Intensity) and CPET determination. Determination from the acoustic analysis was in good agreement with the results from CPET system, where discrepancy of *V̇*O_2_ was within *4* mL/min/kg and the standard error of the mean was *0.39* mL/min/kg (Fig. 3). The error rate of *V̇*O_2_ was less than *20%* for most cases (Table 1).

**Fig. 4:**
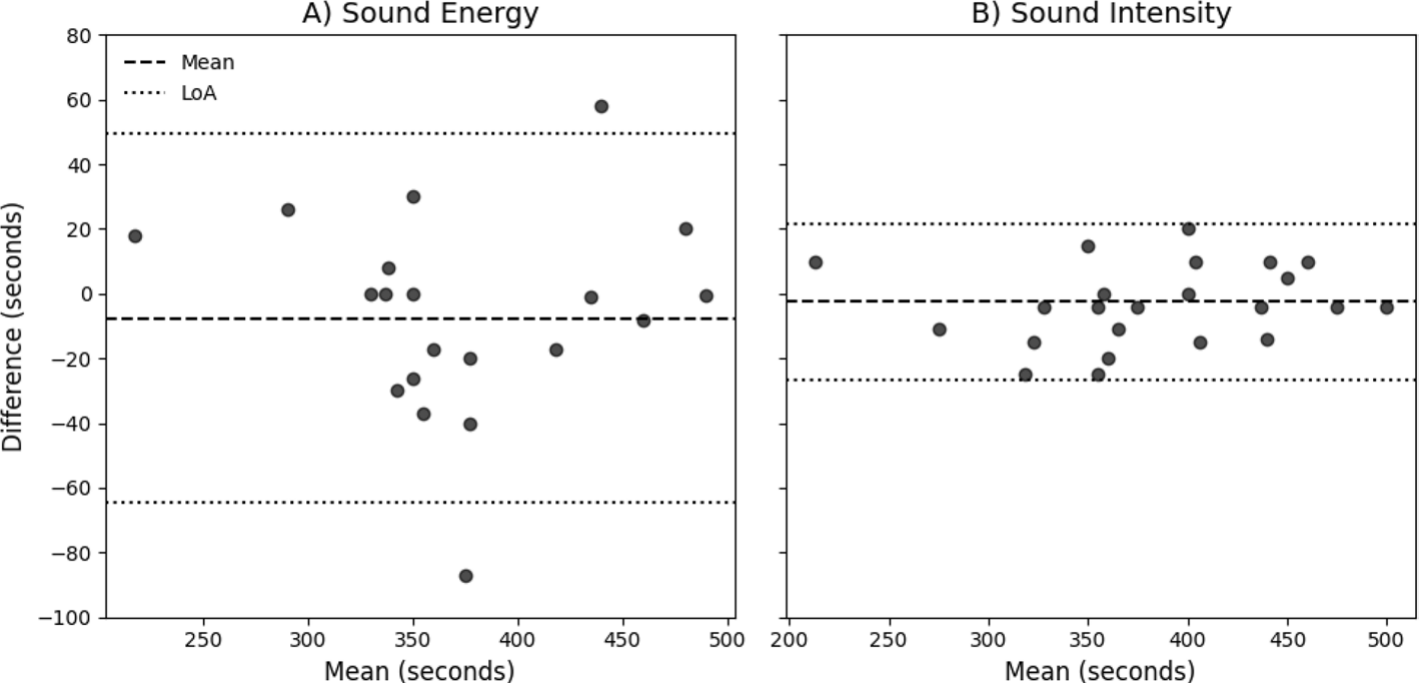
Bland–Altman analysis of the agreement between the ventilatory AT time determined by the CPET system and the acoustic features. **A** Agreement using sound energy. **B** Agreement using sound intensity. The dashed horizontal line indicates the mean difference (bias), and the dotted lines indicate the 95% limits of agreement. Both plots are presented on a shared vertical axis range

Figure 4 shows the Bland–Altman plots for comparing the determined ventilatory AT time between the CPET values and estimated values based on acoustic features. The mean and standard deviation (SD) of time difference between the CPET and estimate values were − *7.62* $\pm$ *29.06* s and − *2.50* $\pm$ *12.25* s, using sound energy and sound intensity, respectively. The 95% confidence intervals (CI) were − *64.57* to *49.33* s (sound energy) and − *26.50* to *21.50* s (sound intensity).

### ***VO***_***2peak***_*** estimation***

**Fig. 5:**
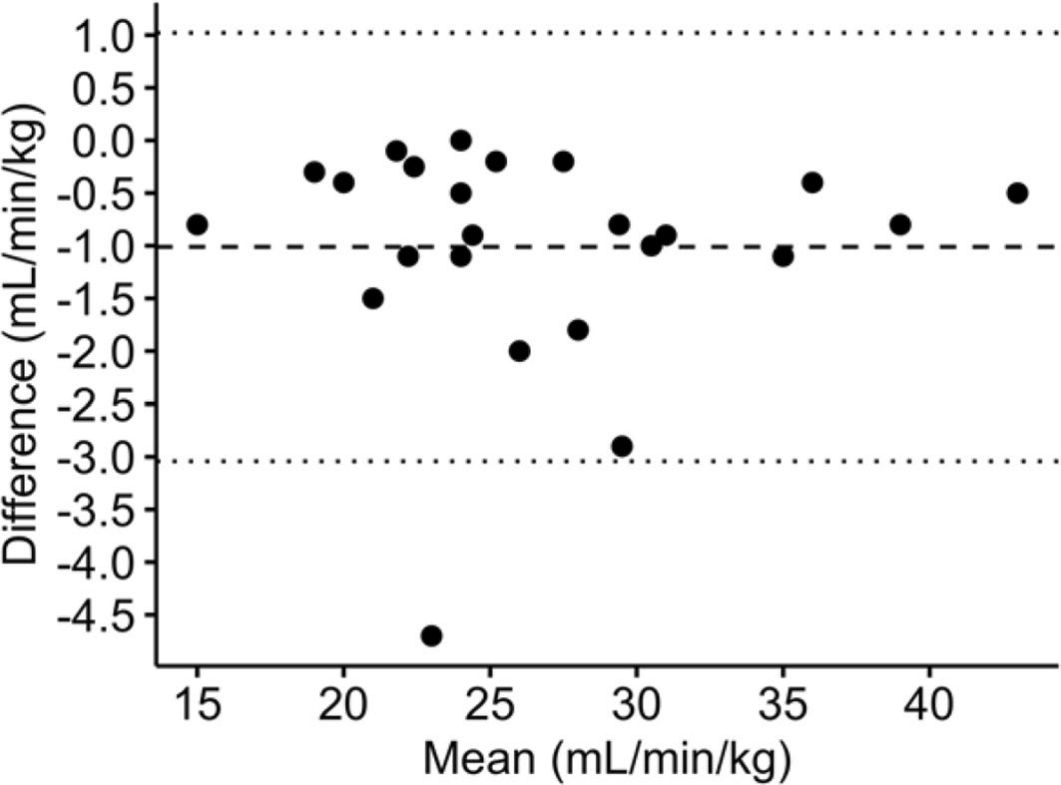
Bland–Altman analysis of VO_2peak_ measured by the CPET system and estimated by random forest for subject-specific model

**Fig. 6:**
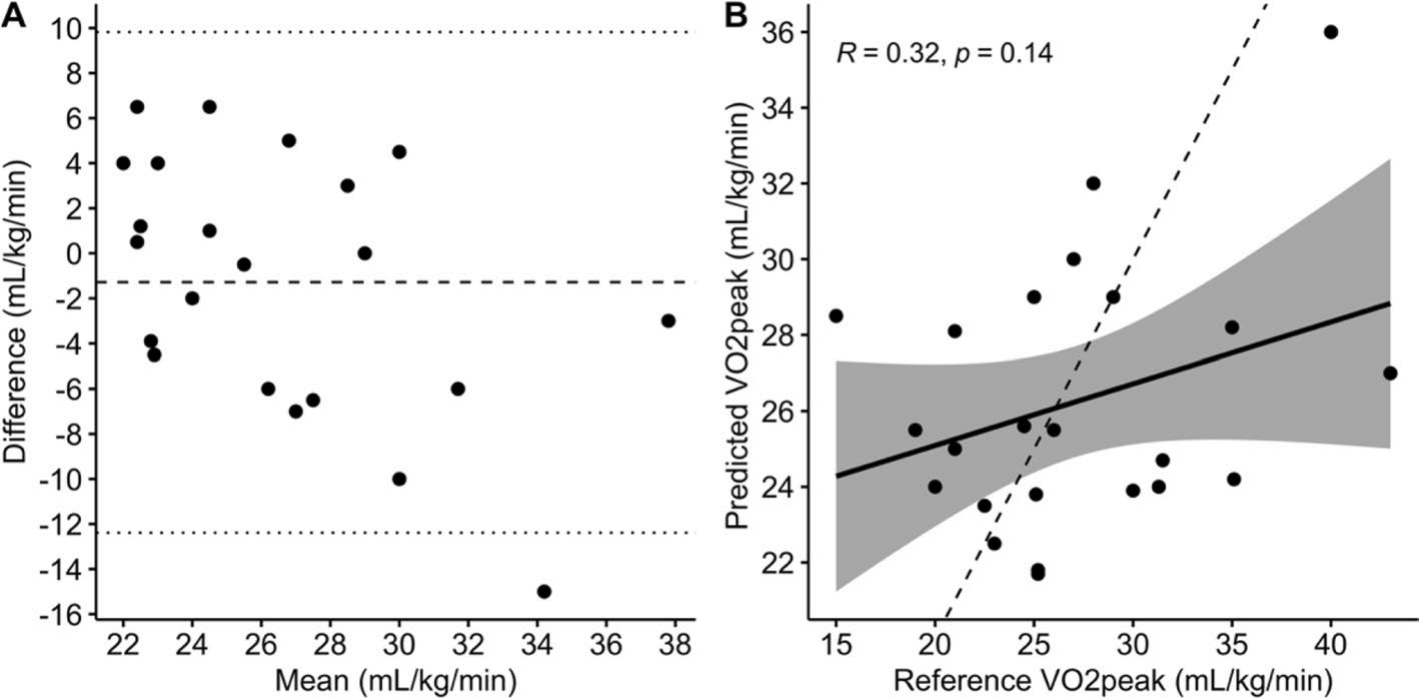
Demonstration of the **A** Bland–Altman analysis and **B** correlation plot of VO_2peak_ measured by the CPET system and estimated by the subject-independent validation approach

In Fig. 5, the subject-specific model estimated VO_2peak_ with *95%* CI of − *0.95* to *0.96* mL/kg/min and the difference of − *1.00* $\pm$ *1.02* mL/kg/min (mean $\pm$ SD). On the other hand, the subject-independent validation model using random forest yielded a wider *95%* CI of − *13.34* to *11.81* mL/kg/min (Fig. 6A) and VO_2peak_ difference of *0.77* $\pm$ *6.42* mL/kg/min. Pearson correlation analysis gave a weak correlation coefficient of *0.32* between the predicted and measured VO_2peak_ with a *p* value of *0.14* (Fig. 6B).

**Fig. 7:**
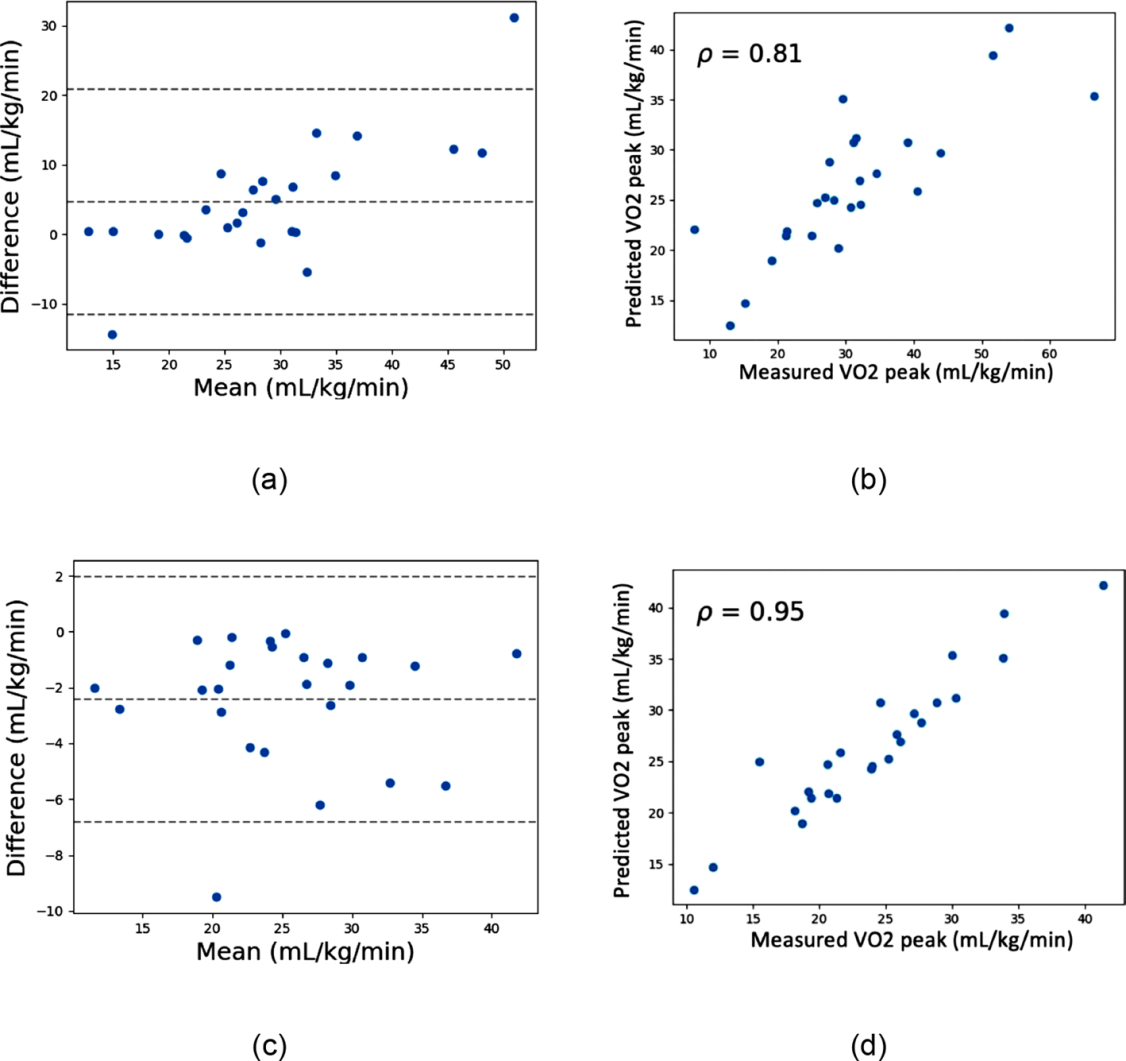
Models trained with RR, sound energy, and RR × intensity. **a** Bland–Altman plot of MLR, **b** scatter plot of predicted vs. measured VO_2peak_ using MLR, **c** Bland–Altman plot of random forest, and **d** scatter plot of predicted vs. measured VO_2peak_ using random forest

To further investigate the model behavior, additional experiments were performed using predefined feature sets. When only respiratory features (respiratory rate, tracheal sound energy, and RR × tracheal sound intensity) were included, multiple linear regression (MLR) predicted VO_2peak_ with a 95% CI of − 11.57 to 20.91 mL/kg/min and correlation coefficient of *r* = 0.81 (Fig. 7A, B). In comparison, the random forest (RF) model achieved substantially improved performance with a narrower CI of − *6.95 to 2.05* mL/kg/min and stronger correlation (*r* = *0.95*, Fig. 7C, D). Bland–Altman analysis confirmed that RF slightly underestimated VO_2peak_ but within smaller limits of agreement compared to MLR.

**Fig. 8:**
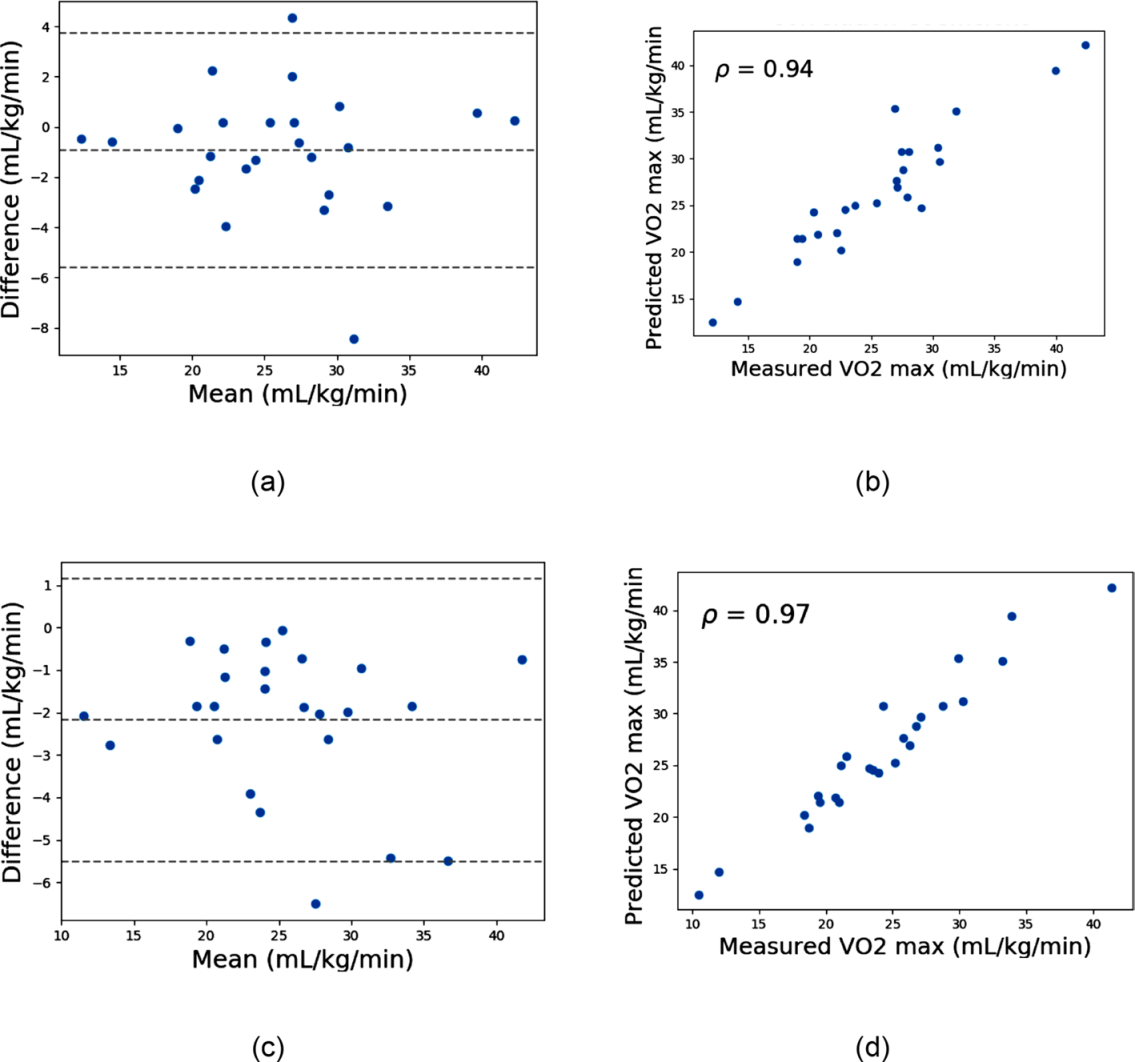
Models trained with HR, RR, tracheal sound energy, RR × tracheal sound intensity, and workload. **a** Bland–Altman plot of multiple linear regression (MLR), **b** scatter plot of predicted vs. measured VO_2peak_ using MLR, **c** Bland–Altman plot of random forest, and **d** scatter plot of predicted vs. measured VO_2peak_ using random forest

When HR and workload were added to the respiratory features, MLR performance improved, narrowing the CI to − 5.00 to 3.74 mL/kg/min and yielding *r* = *0.94*. However, RF remained superior, with CI = − 5.44 to 1.25 mL/kg/min and *r* = *0.97* (Fig. 8).

## Discussion

This study aimed to show proof of concept for testing the application of a convenient and cost-effective wearable device (*The Patch*) in assessing exercise capacity. The primary finding of this study is that of tracheal sound analysis, specifically sound intensity can estimate ventilatory AT during submaximal exercise. In addition, we demonstrated the feasibility of using these sounds to estimate VO_2peak_.

The ventilatory response to metabolic acidosis provides the physiological foundation for using tracheal sounds to estimate AT [36, 37]. The breakpoints and changes in the slopes of VCO_2_/VO_2_, ventilatory equivalents V_E_/VO_2_ and V_E_/VCO_2_ are used for determining ventilatory AT by ventilatory method [4, 11]. At the AT, the buffering of lactic acid generates excess CO_2_. This drives a non-linear increase in minute ventilation (V_E_) to expel the CO_2_. Since tracheal sound intensity is known to correlate linearly with airflow and turbulence, the ‘breakpoint’ in ventilation physically manifests as a ‘breakpoint’ in sound intensity. This physical link explains why our acoustic method tracked the physiological AT with 91.7% temporal accuracy. Those breakpoints were used to determine ventilatory AT.

When comparing acoustic features, sound intensity established more robust performance than sound energy. We observed that ventilatory AT detection failed only in 4% of participants using sound intensity, compared to 16% of participants using sound energy. These detection failures may primarily be driven by breathing pattern irregularities during the early stages of the stepwise protocol (Fig. 11). Specifically, erratic breathing or hyperventilation at the test onset might have created high-amplitude ‘local peaks’ in sound energy. These artifacts potentially masked the true physiological breakpoint, making distinct ventilatory AT identification impossible in those specific cases. In contrast, sound intensity, which accounts for breath duration, was less susceptible to these initial fluctuations, and led to superior detection rate.

Clinically, the accuracy of determining the ventilatory AT workload is a critical finding for exercise prescription, as ventilatory AT demarcates the upper limit of a range of exercise intensity that can be accomplished predominantly aerobically [13]. In this study, the ventilatory AT workload determined by sound intensity and sound energy achieved 100, and 88% agreement with the CPET system, respectively (Fig. 2 and Table 1). The high accuracy in determining ventilatory AT workload by our wearable device (*The patch*) demonstrates its potential of wide use by professionals prescribing aerobic exercise for healthy subjects and subjects with cardiorespiratory diseases, without need of complex system and apparatus.

To the best of our knowledge, this is the first study using acoustic signals of breathing sounds to predict VO_2peak_. We developed two machine learning approaches for VO_2peak_ prediction: a subject specific model and a subject independent validation.

Our subject specific model demonstrated high performance, with narrow limits of agreement and high accuracy for VO_2peak_ prediction (Fig. 5). This proposed model is customized for each individual and its ultimate use is to allow estimating VO_2peak_ using data at lower exercise stages so as to reduce the risks associated with high intensity exercise. Therefore, the generalizability of subject-independent model is affected by the nature that each model is developed for every individual. However, this model requires the measurements from the CPET system. This approach could be used for VO_2peak_ follow-up evaluations in centers, where the CPET system is available. For instance, a patient could undergo one baseline CPET to train their personal model, and subsequent assessments could be performed using only the wearable device. This would reduce the duration and stress of testing, as maximal effort would not be required for every follow-up. Moreover, this subject specific model for VO_2peak_ prediction can be accounted as an alternative for cardiac patients, whose maximal and sub-maximal efforts are not advisable due to adverse events risks [38].

For the subject-independent validation (leave-one-subject-out cross-validation), performance depended heavily on the feature set. The direct random forest approach initially showed modest predictive ability, with wider CI and a lower correlation with measured VO_2peak_ (*r* = *0.32*). This highlights the challenge of applying a one-size-fits-all model across individuals. However, when subject-independent models were systematically trained using predefined feature sets (respiratory rate, sound energy, and acoustic ventilation), predictive accuracy improved markedly (*r* = *0.95*), outperforming multiple linear regression (*r* = *0.81*) (Fig. 7). Further inclusion of heart rate and workload enhanced performance, with random forest reaching a correlation of *r* = *0.97* (Fig. 8). These results imply that although acoustic signals have a great deal of predictive ability, the most reliable and broadly applicable estimation is produced when they are combined with fundamental cardiac and workload measures.

A key strength of our methodology was the sensor location. Regarding sensor attachment, the suprasternal notch was selected not only for its proximity to the trachea but also for its anatomical stability. Unlike the chest wall or extremities, which experience significant skin deformation and muscle contraction during cycling, the suprasternal notch remains relatively stable [29], which also minimizes mechanical strain on the adhesive interface. Furthermore, unlike ECG or bio-impedance sensors, where signal integrity relies on constant electrical conductivity (which is highly sensitive to sweat and hydration changes) [39], the contact microphone relies on mechanical coupling. While perspiration remains a challenge for any skin-contacting wearable, the combination of this stable anatomical location and the use of robust double-sided medical adhesive helps mitigate detachment issues.

Several limitations of this study must be acknowledged. First, the study was conducted using a cycle ergometer to minimize background noise compared to a treadmill. Future research is needed to test the accuracy of our device in high-noise clinical settings and on treadmills, where footfalls create significant acoustic interference.

Second, the stepwise exercise protocol may have introduced artifacts. The sudden increase in resistance every 2 min likely induced transient spikes in ventilation as participants acutely adjusted to the new load. Future studies should validate *The Patch* using a ramp exercise protocol, where work rate increases continuously. We hypothesize this would induce a smoother, linear increase in physiological demand, minimizing transient ventilatory adjustments and yielding a cleaner sound energy signal.

Third, a limitation of this study is the submaximal nature of the protocol (stopped at 85% HR). Therefore, our VO_2peak_ prediction models should be interpreted as estimating the ‘peak attained’ in a submaximal context. Future studies using maximal-effort protocols are needed to validate the model for true VO_2max_ prediction.

Finally, regarding the study population, our sample size was small (*n* = *24*) and predominantly female (*75%*). Due to the limited number of male participants (*n* = *6*), we were unable to perform a robust statistical analysis to detect potential sex-based differences in tracheal sound characteristics. While sex is a known determinant of exercise capacity [29], future studies with larger, balanced cohorts are required to investigate if acoustic features differ between sexes. In addition, the leave-one-subject-out cross-validation evaluates the subject-independent performance of the modeling approach, but does not produce a single population model. The current results suggest the potential for a generalized population model, which would need to be confirmed in a larger data set or in an external validation cohort.

## Conclusion

This study demonstrates that tracheal sound analysis, specifically sound intensity is a robust method for identifying the ventilatory AT. Our results showed that sound intensity achieved a 96% detection rate and 100% agreement in workload determination compared to the CPET system. While the estimation of VO_2peak_ was found to be feasible, particularly using subject-specific models, the subject-independent validation model requires further validation in maximal-effort protocols to address the submaximal limitations of this study. Overall, these findings highlight the potential of the wearable device (*The Patch)* as a cost-effective, non-invasive tool for potential exercise prescription and functional capacity monitoring in settings, where traditional CPET is unavailable.

## Methods

### Participants

Healthy adults aged between 18 and 65 years with no history of cardiovascular or pulmonary disorders were recruited in this study. Healthy adult participants were chosen to minimize the likelihood of clinically relevant abnormalities and to ensure the ability to sustain higher exercise intensities across a wider physiological range. Individuals who were allergic to medical tape or with any disability precluding physical exercise were excluded. The study protocol was approved by the Research Ethics Board (REB), University Health Network, Canada (REB#16-5167). All participants signed an informed written consent before participation in the study.

### Measurements

**Fig. 9:**
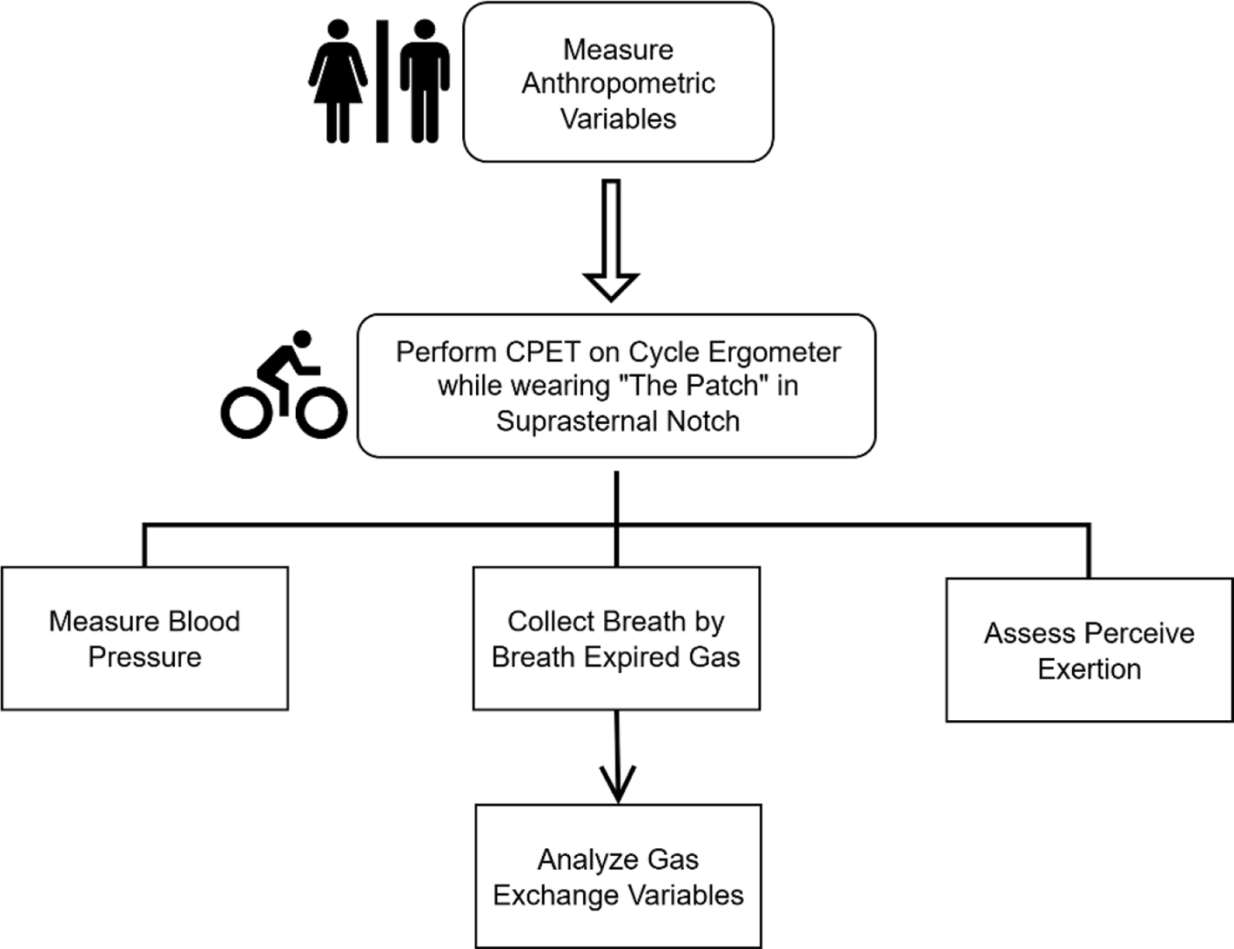
Schematic diagram of measurements taken during the study

The study was conducted in the SleepdB laboratory at KITE—Toronto Rehabilitation Institute, University Health Network. We recorded anthropometric variables, including height, body mass, resting blood pressure (sitting position), and physical activity level using Baecke’s questionnaire [40]. Then, the participant performed the submaximal stepwise incremental CPET protocol on cycle ergometer. Cycle ergometer was chosen, because it provides more accurate measurement of exercise capacity compared to the arm ergometer, and it is less prone to motion artefacts and noises compared to the treadmill test [12]. During the CPET test, blood pressure was measured every 2 min at the end of each exercise stage using a manual aneroid sphygmomanometer [41]. Participants breathed through a facial mask to collect breath-by-breath expired gas. The gas exchange analysis system (Vmax Encore V229, CareFusion, San Diego, CA, US) was used to measure ventilatory and metabolic gas exchange variables [11, 13]. Ventilatory and metabolic parameters were continuously recorded, and perceived exertion was assessed at the end of each stage using the modified Borg Rating of Perceived Exertion (0–10 scale) [4, 42]. A schematic diagram of measurements taken during the study is shown in Fig. 9.

**Fig. 10:**
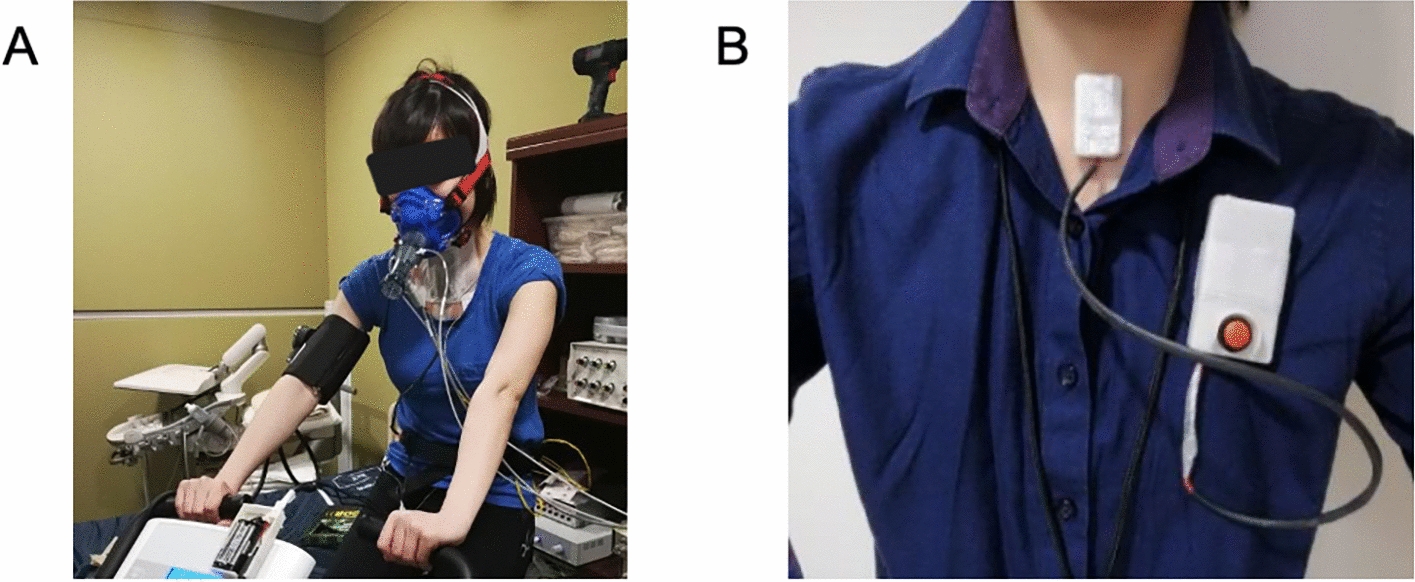
**A** Experiment setup of the exercise test, **B** placement of *The Patch* at the suprasternal notch

Simultaneous with CPET recording, tracheal respiratory sounds were acquired using a wearable device (*The Patch*) attached to the suprasternal notch with double-sided medical adhesive (Fig. 10B). *The Patch* incorporated a unidirectional microphone (sampling frequency: 15,277 Hz) and stored signals in onboard memory. The experimental setup and device placement are shown in Fig. 10A.

### The exercise protocol

The CPET started with a 3-min warm up at a pedaling speed of 60 revolutions per minute (rpm). Every 2 min, the resistance was increased to change the workload. The workload increment was individualized, calculated using modified Wasserman’s Equation [4]. Wasserman’s equation was developed for 1-min exercise incremental stages, however, as shown in Equation S1 (Supplementary document), we doubled the incremental workload to a 2-min stepwise protocol to have enough time for stabilizing physiological signals and measure respiratory and HR. Following peak exercise, participants completed a 2-min active recovery period.

**Fig. 11:**
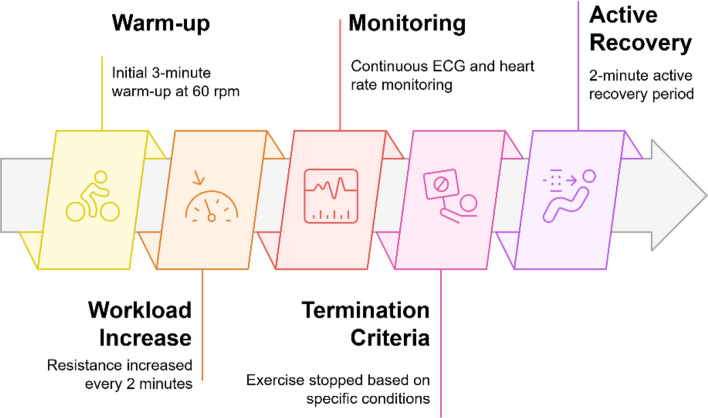
Schematic diagram of the exercise protocol of the study

During CPET (Fig. 10A), ECG and HR were continuously monitored as recommended by American Heart Association (AHA) [3, 11, 13]. Exercise test was terminated if participants: (1) reached 85% of their maximal HR (220 minus age), (2) developed excessive blood pressure responses (systolic > 250 mmHg or diastolic > 115 mmHg); (3) experience volitional exhaustion; or (4) demonstrated ECG abnormalities or reported adverse symptoms (e.g., chest pain, dyspnea, dizziness, and lightheadedness). Thus, the CPET was performed to a submaximal level (≤ 85% of predicted maximal HR). A schematic of the exercise protocol is shown in Fig. 11, with a detailed stage breakdown provided in Supplementary Table S1.

### Gas exchange analysis

The CPET gas exchange analysis system (V_max_ Encore V229, CareFusion, San Diego, CA, US) was used to measure ventilatory and metabolic gas exchange variables. Ventilatory method was used to determine the gold standard AT. Two independent expert evaluators manually determined AT using a combination of V-slope method, metabolic variables and ventilatory equivalents (V_E_/VO_2_, V_E_/VCO_2_) [4]. If there was a difference of > 20 s, a third evaluator assessed the AT independently.

The reference value of VO_2peak_ was determined by gas exchange, where breath by breath oxygen uptake (VO_2_) was averaged every 10 s and the peak value was chosen as VO_2peak_. It is noted that due to the termination criteria (85% of predicted max HR), the measured VO_2peak_ represents a symptom-limited or protocol-limited peak, rather than a physiological VO_2max_. Consequently, our analysis focuses on the robust physiological breakpoint of AT, with VO_2peak_ estimation treated as a feasibility investigation.

### Acoustic analysis

**Fig. 12:**
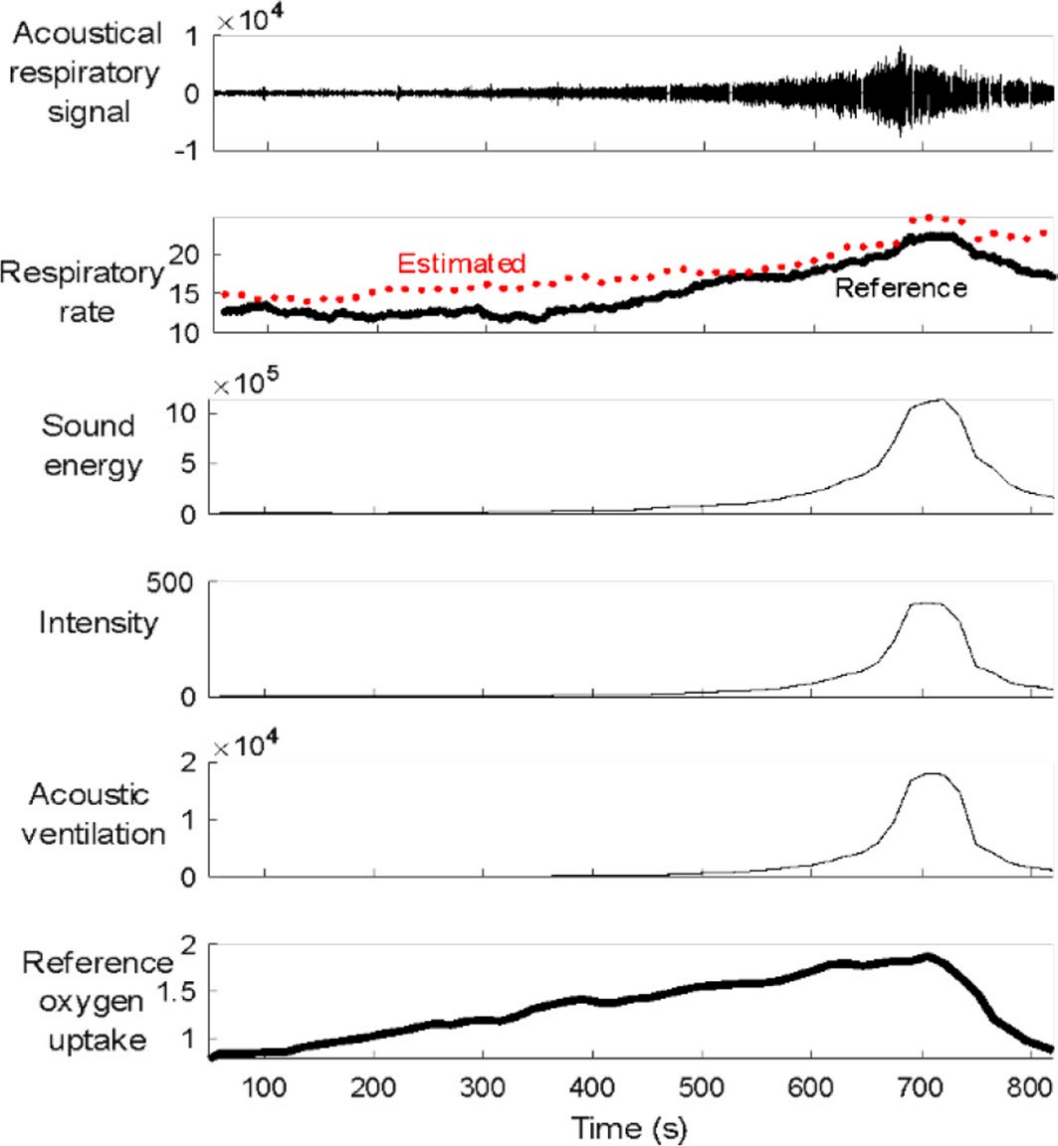
Example trace of respiratory sound extracted from recorded tracheal sound during CPET, in addition to extracted features, and reference oxygen uptake from the CPET system

The sound signals were processed using MATLAB (2018b, MathWorks, Natick, MA) software. Since the exercise intensity was incremented for sequential exercise stages, the signals were segmented based on exercise stages. The sounds were bandpass filtered between 100 to 3000 Hz, the typical frequency range of normal tracheal respiratory sounds [43, 44]. Then, the filtered sounds were down-sampled to 6000 Hz to save computational cost. To remove ambient noise from the filtered sound, synchrosqueezing transform, which was successfully evaluated in our previous study [35], was used. To remove motion artefacts, an adaptive spike removal algorithm was applied subsequently. The extracted filtered sounds includes the respiratory-related sounds, deemed respiratory sounds. For each exercise stage, the respiratory sound was analyzed to detect respiratory phases using a previously validated algorithm [33]. From respiratory sounds, four features, including sound energy, sound intensity, respiratory rate and acoustic ventilation were extracted (Fig. 12). For more information about the features and their association to the metabolic variables see supplementary document.

### Anaerobic threshold (AT) estimation

To determine ventilatory AT from sound-related features, we hypothesized that the breakpoints in the traces of the features in terms of time occurred at the physiological ventilatory AT. The nadir of ventilatory equivalents (V_E_/VO_2_, V_E_/VCO_2_) were parameters we used for reference ventilatory AT determination using ventilatory method [4]. These breakpoints were manually marked by two independents assessors (CF and SK). See more details in the supplementary document.

### ***Peak oxygen uptake (VO***_***2peak***_***) estimation***

In addition, we investigated the association between sound related features and the VO_2_ measured by the CPET system. To estimate VO_2_ from the sound related features, we developed two machine learning models. We used regression random forest, which implements regression analysis using decision trees [45, 46]. All the sound related features were used as independent variables in the Random Forest linear regression model [46] to predict VO_2_. To train the model, VO_2_ signal was smoothed every 1 s to remove abrupt noisy artifacts. Moreover, two training approaches were implemented: 1 subject-specific and 2 subject-independent validation approaches. In subject-specific approach, the data of each subject was divided in fivefold. Then, VO_2_ related to each fold was predicted using the model trained by the other folds (fivefold cross-validation) in the same subject. For the subject-independent approach, VO_2_ of a subject was estimated by the model which was trained by the data of other subjects in the study population (leave-one-subject-out cross-validation). For subject-specific and subject-independent approaches, the peak of the estimated VO_2_ was detected for each subject as VO_2peak_.

### Statistical analysis

Statistical analysis was performed using R version 3.4.0. The agreement between correlations between the respiratory intensity and VO_2_, VCO_2_, Tidal Volume (V_t_), and V_E_ from the CPET measurement was assessed using Pearson correlation. The exercise stage (i.e., workload) at which estimated ventilatory AT occurred and the predicted VO_2peak_ were compared to the reference parameters using Bland–Altman analysis. Successful ventilatory AT detection was defined as ventilatory AT for both methods occurring at the same exercise stage. Pearson’s correlation coefficients were also used to quantify the agreement between estimated VO_2peak_ using subject specific and subject-independent models and reference values.

## Supplementary Information

Supplementary Material 1.

## Data Availability

All data generated or analyzed during this study are included in this published article **.**
