# Supplementary material for: Estimation of anaerobic threshold and peak oxygen uptake from tracheal sound during cycle ergometer cardiopulmonary exercise test
Source: Biomed Eng Online. 2026 Feb 27;25:55. doi: 10.1186/s12938-026-01540-7 (PMC13049783; doi:10.1186/s12938-026-01540-7)
Supplement: Supplementary file 1 — Supplementary Material 1. [file 12938_2026_1540_MOESM1_ESM.docx]

Supplementary Document

**Estimation of Anaerobic Threshold and Peak Oxygen Uptake from Tracheal Sound during Cycle Ergometer Cardiopulmonary Exercise Test**

Qi Zhang, Nasim Montazeri Ghahjaverestan , Cristina de Oliveira Francisco, Muammar Muhammad Kabir, Md. Saiful Hoque, Shahram Kharabian Masouleh, Paul Oh, Susan Marzolini, Azadeh Yadollahi

**The Exercise Protocol**

${Ramp}_{ind}\frac{(w}{min)}$ = $2 \times\left( (height,cm-age, years \right) \times20 for sedentary men and \times14 for sedentary women-(150+\left( 6 \times weight, kg \right))/100$……………………….(**Eq. S1**)

**Table S1**: Cycle ergometer exercise test protocol for healthy participants

| **Period** | **Time (min)** | **Workload (watts)** | **Exercise Stages** |
| --- | --- | --- | --- |
| Warmup | 0 – 3 | Minimum | Warmup |
| 12-minute testing | 3 – 5 | ${Ramp}_{ind}$ | Exercise stage 1 |
|  | 5 – 7 | 2 × ${Ramp}_{ind}$ | Exercise stage 2 |
|  | 7 – 9 | 3 × ${Ramp}_{ind}$ | Exercise stage 3 |
|  | 9 – 11 | 4 × ${Ramp}_{ind}$ | Exercise stage 4 |
|  | 11 – 13 | 5 × ${Ramp}_{ind}$ | Exercise stage 5 |
|  | 13 – 15 | 6 × ${Ramp}_{ind}$ | Exercise stage 6 |
| Recovery | 15 – 17 | minimum | Active recovery |
|  | 17 – 25 | none | Passive recovery |

***Acoustic Analysis***


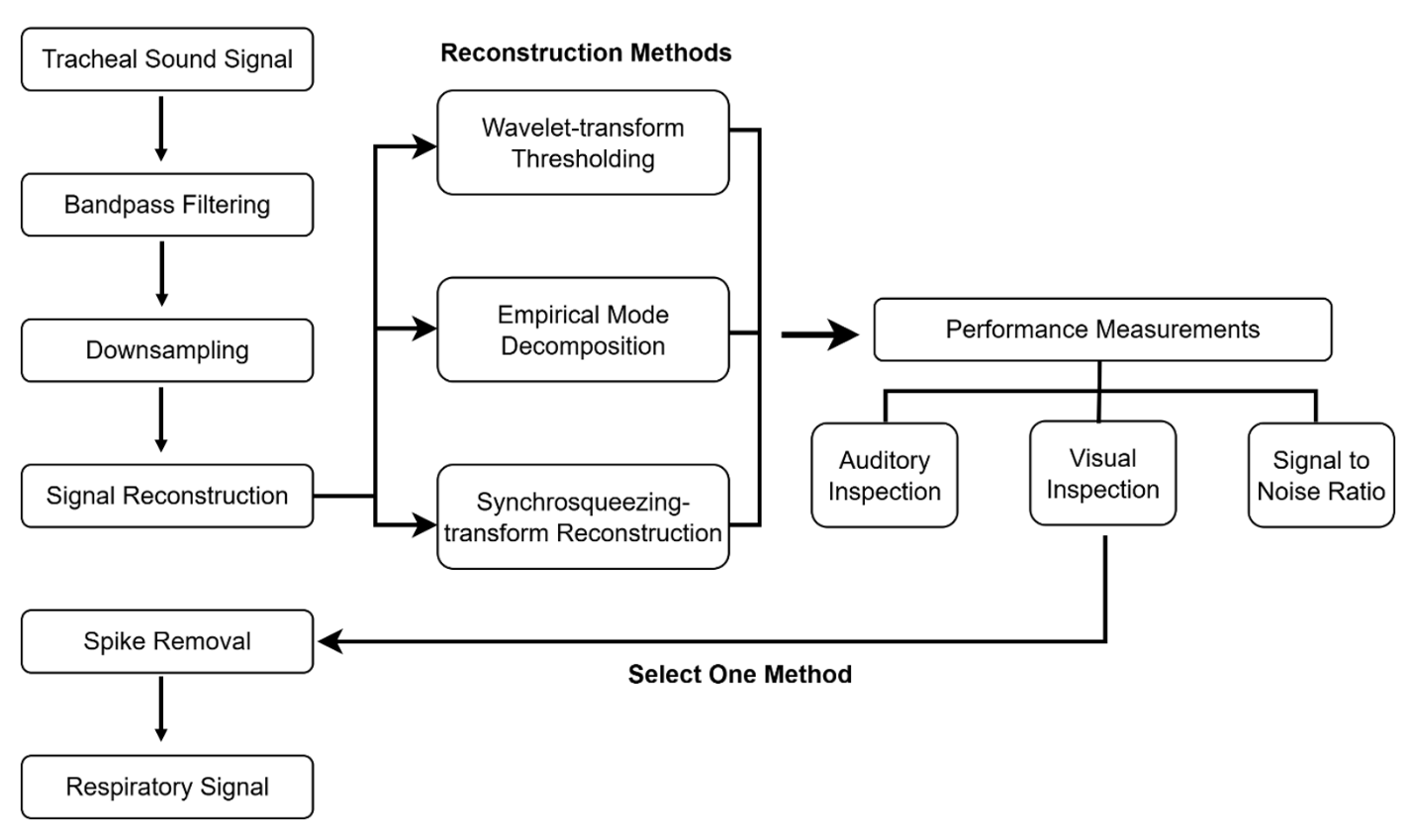


**Figure S1**: Schematic diagram of cardio-respiratory signal retrieval

***Extracted sound features***

Sound energy was calculated as the sum of squared amplitudes of the respiratory sound signal during each inspiration. Sound intensity was then defined as the sound energy divided by the duration of the inspiration phase (seconds). Then, respiratory rate (RR_Patch_) was calculated based on the distance of consecutive breaths. The acoustic ventilation was defined as sound intensity multiplied by RR_Patch._ Then, the features were smoothed using a sliding window of 60 seconds with 50 seconds overlap.

***Correlation Analysis***

The correlations were estimated using Pearson’s correlation data and the least square regression analysis was applied to each subject. This step was to validate our hypothesis and set up rationale for the following investigation.

Sound intensity of the respiratory signal recorded at the trachea highly correlates with the metabolic variables (**Figure S2**). Sound intensity and tidal volume were highly correlated (*r = 0.91 ± 0.05*). The promising results indicates that tracheal sound intensity is a potential marker of tidal volume and may be a predictor of VO_2_.

**Figure S2**: Boxplot of the correlation coefficients between respiratory intensity and VO_2_, VCO_2_, V_t_ and V_E_. The red line represents the median value of each box, and the star represents the mean value of all correlation values of the box (Each box shows the maximal, minimal, mean and standard deviation values of the coefficients. Only coefficients of significantly correlated pairs were reported (p < 0.05)). However, to provide a comprehensive view of the data distribution, we present the individual regression plots and raw correlation coefficients for the primary outcome (VO_2_) as a representative example in **Figure S3**.

| 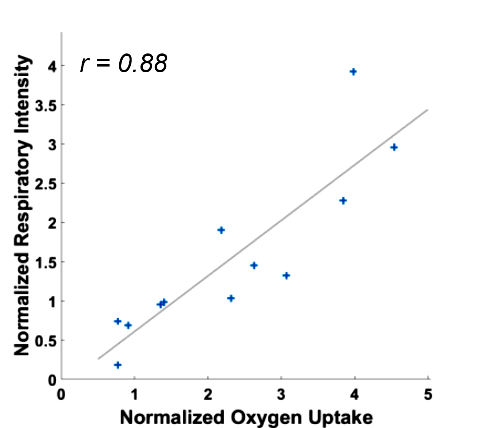 | 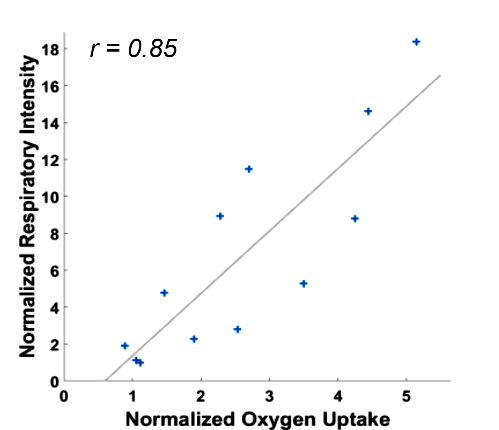 | 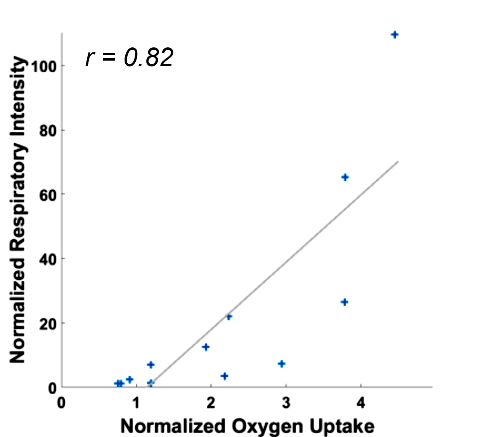 |
| --- | --- | --- |
| 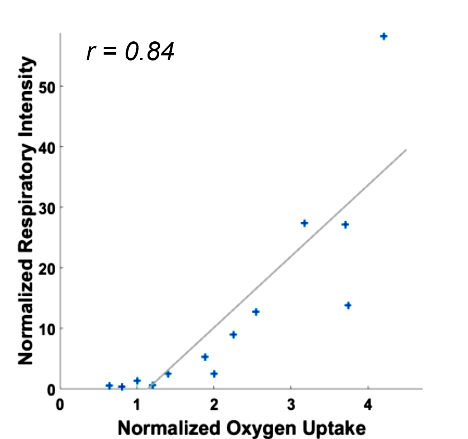 | 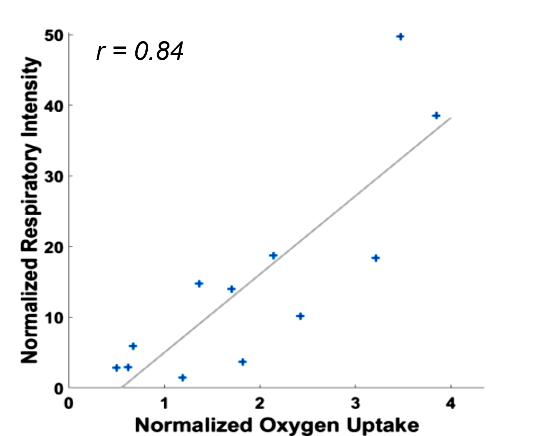 | 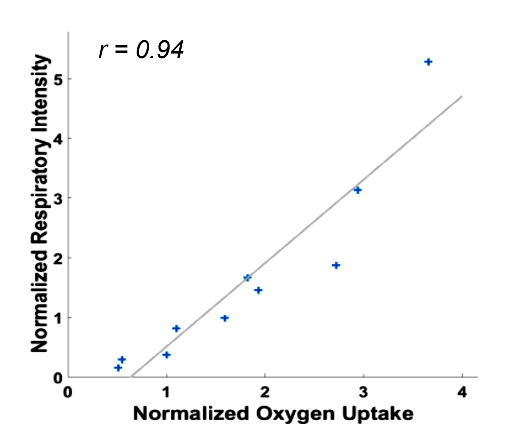 |
| 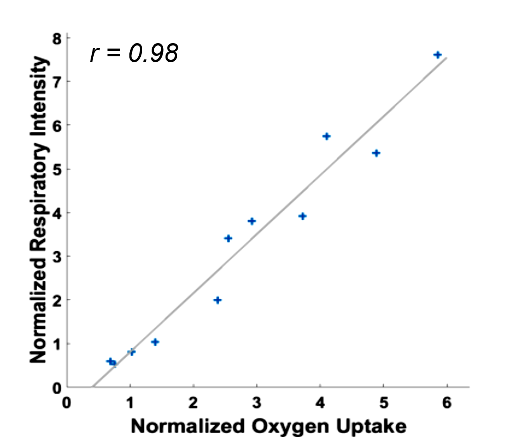 | 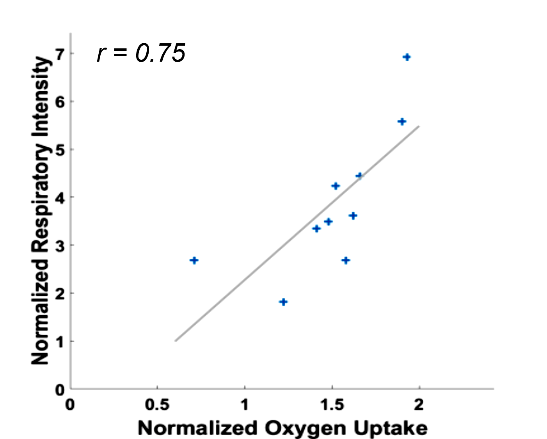 | 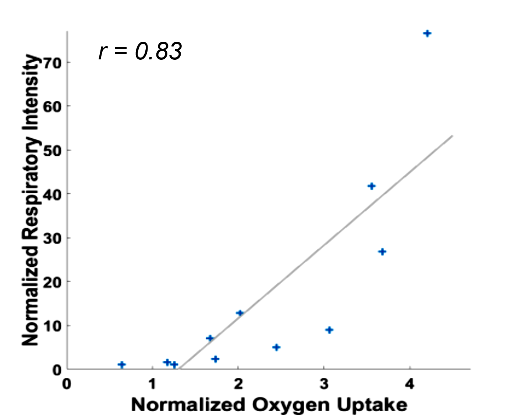 |
| 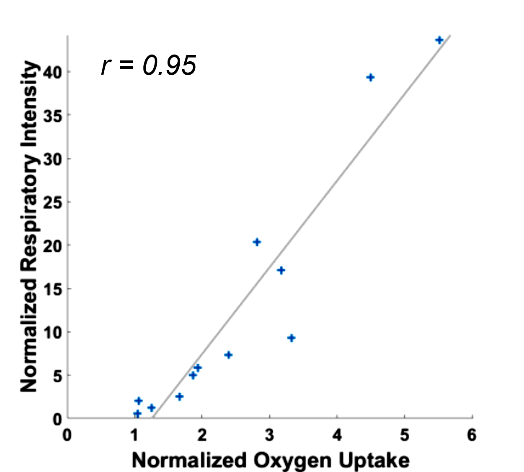 | 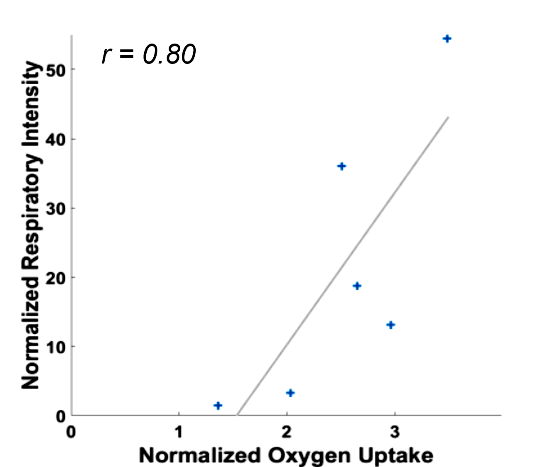 | 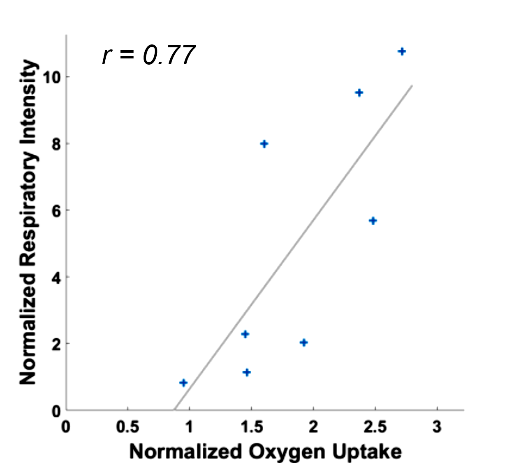 |
| 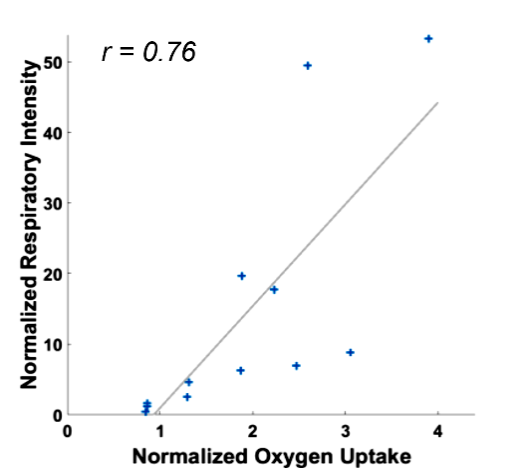 | 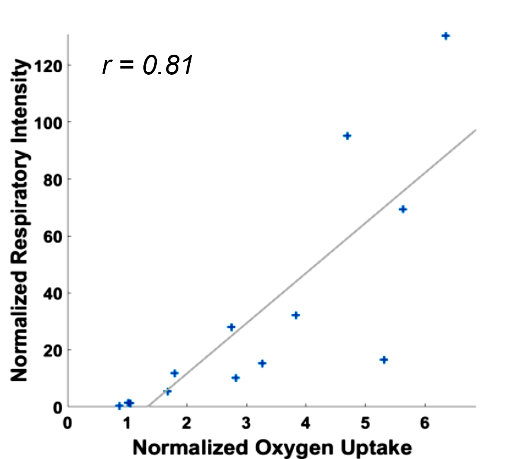 | 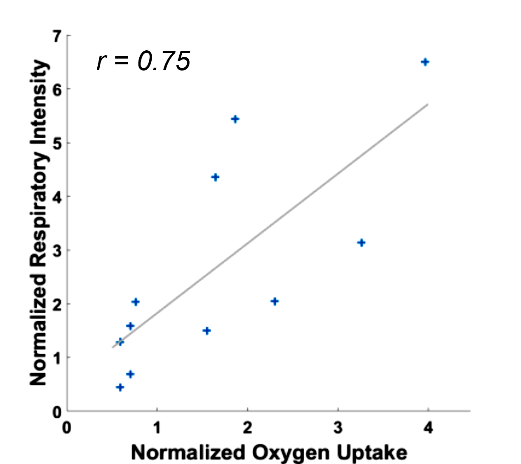 |
| 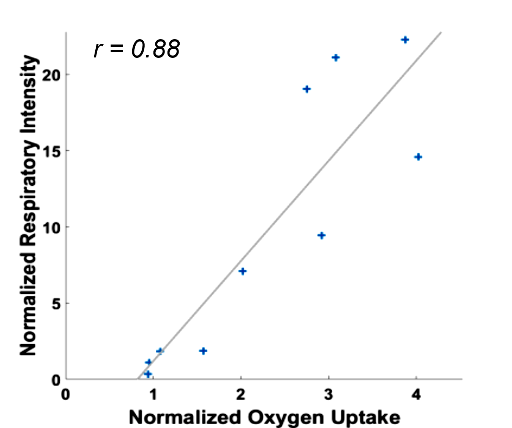 | 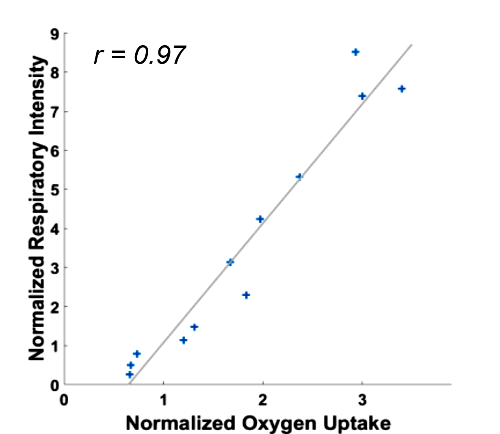 | 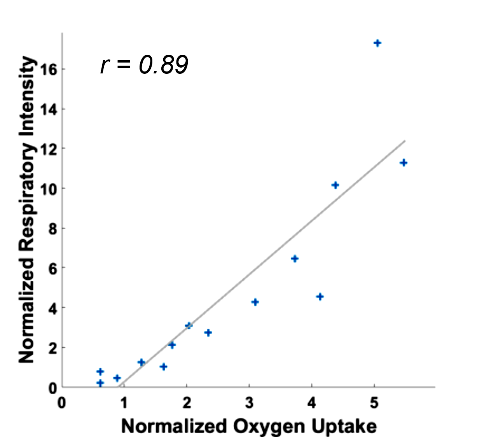 |
| 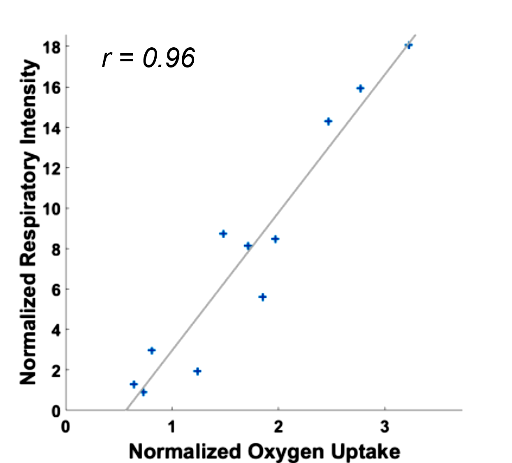 | 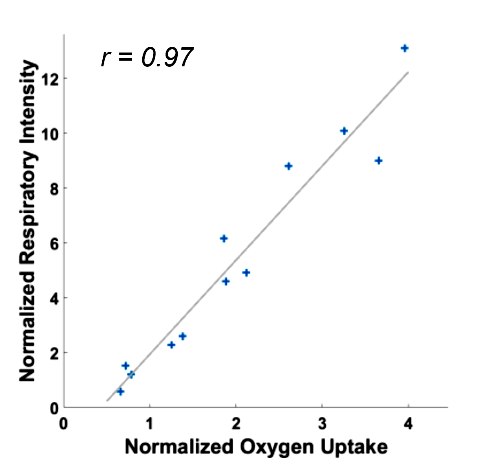 | 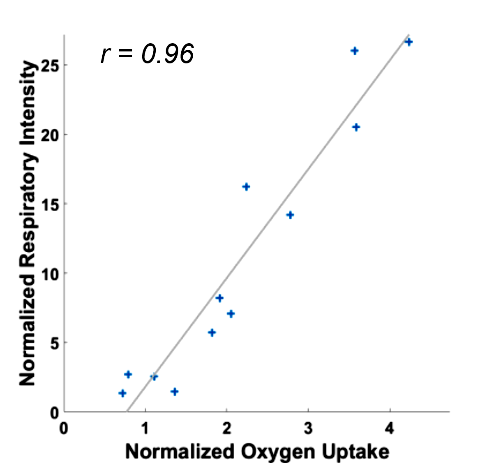 |
| 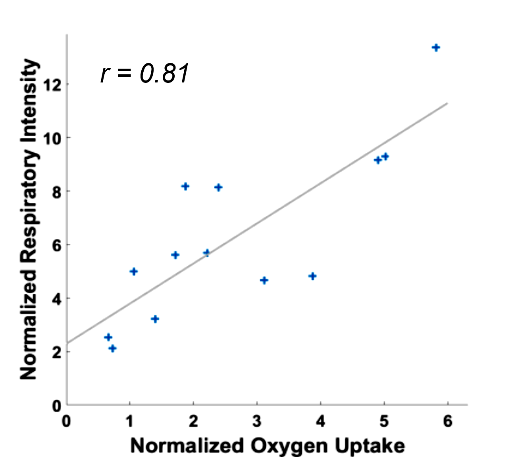 | 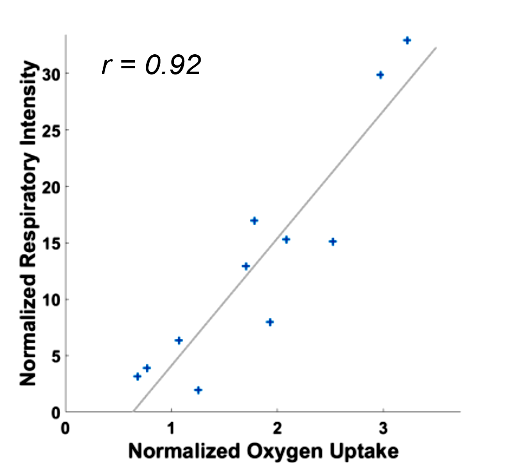 | 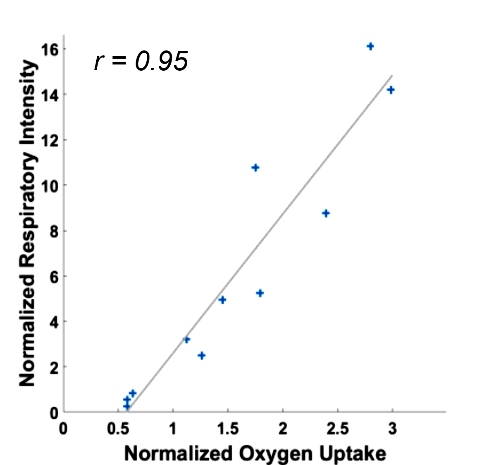 |

**Figure S3**: Individual linear regression analysis of respiratory sound intensity versus oxygen uptake (VO_2_) for all participants (*n = 24*). The scatter plots illustrate the relationship between normalized respiratory intensity and normalized VO_2_ for each subject.

The mean and standard deviation of correlation coefficients among features and between individual feature and output were depicted in **Figure S4** and **S5**. We observed an almost 1:1 linear relationship between sound intensity and its multiplication with RR_patch_, so only one of them was selected. RR_patch_ $\times$ sound intensity had higher correlation than sound intensity. Also, tracheal sound energy had higher correlation with VO_2_ as compared to tracheal sound intensity. Therefore, RR_patch_, tracheal sound energy, and RR_patch_ $\times$ tracheal sound intensity were selected as the respiratory features.

| 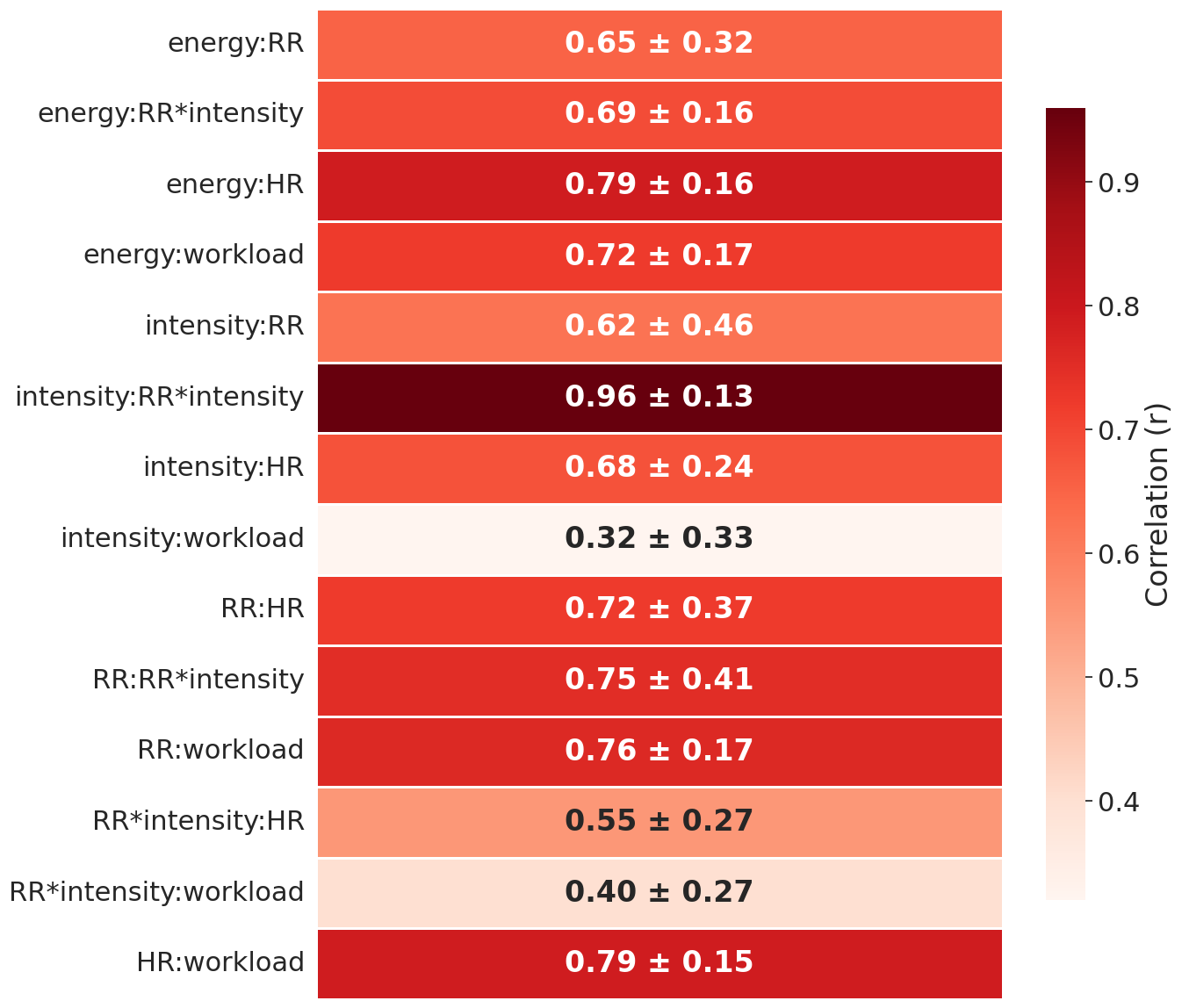 |
| --- |
| **Figure S4**: Heat-map of the Pearson’s correlation coefficients among respiratory features. The color intensity represents the strength of the mean correlation coefficient (r), and the numerical values within each cell report the Mean ± Standard Deviation. |

| 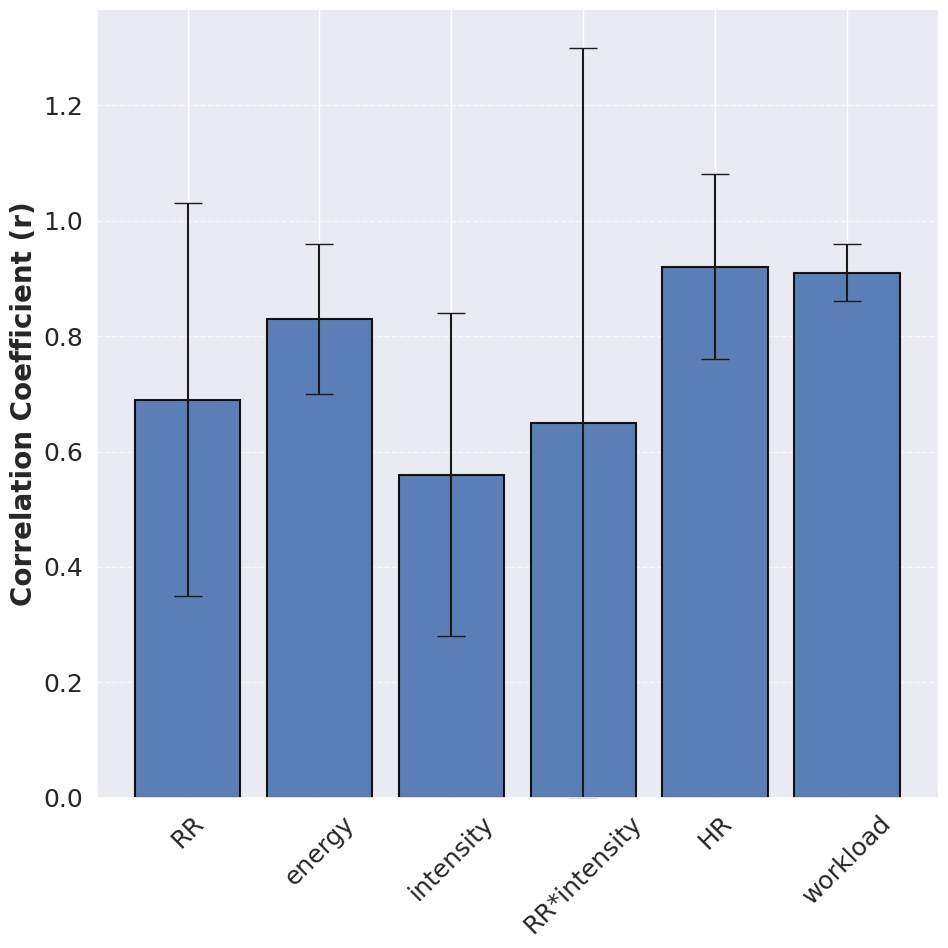 |
| --- |
| **Figure S5**: Pearson’s correlation coefficients between individual features and the reference Oxygen Uptake (VO_2_). The height of each bar represents the mean correlation coefficient (r), and the error bars indicate the standard deviation. |

**Ventilatory *AT Estimation***

By tracing the sound related features over time, breakpoints in the curves were visually detected and compared to the breakpoints observed through CPET system ventilator methods (V-slope method, metabolic variables and ventilatory equivalents). The ventilatory AT determination from CPET system is shown in **Figure S6**.

| (a) | 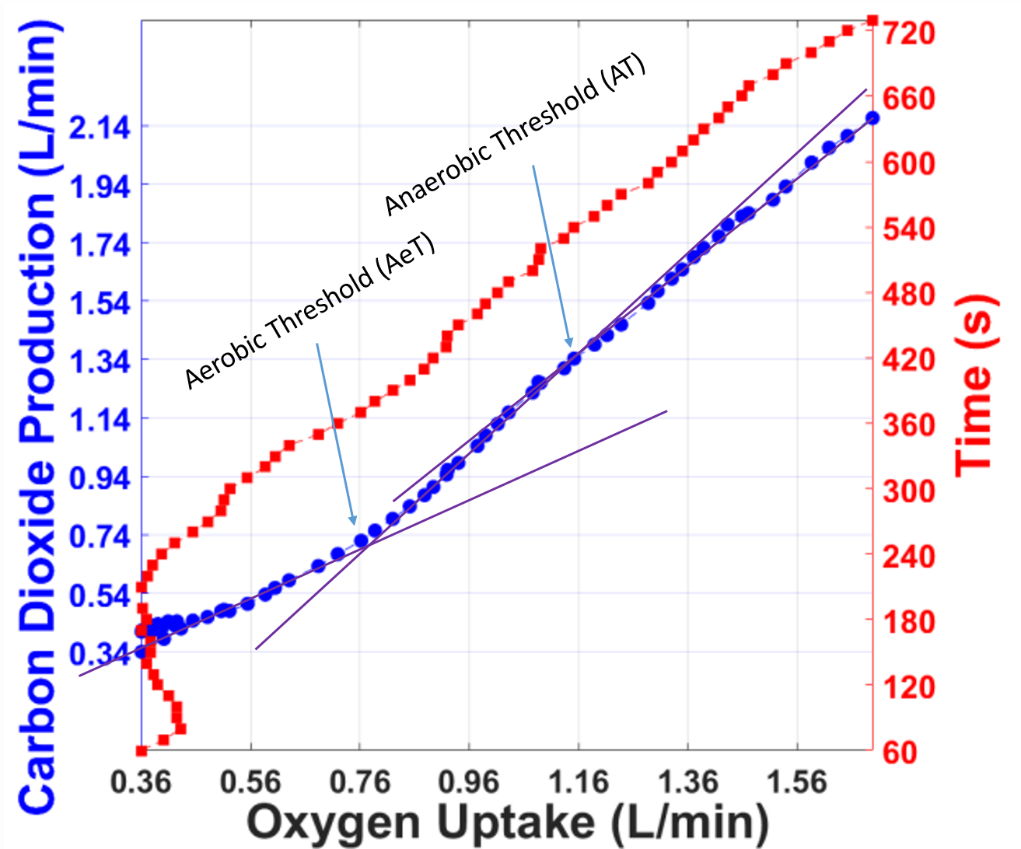 |
| --- | --- |
| (b) | 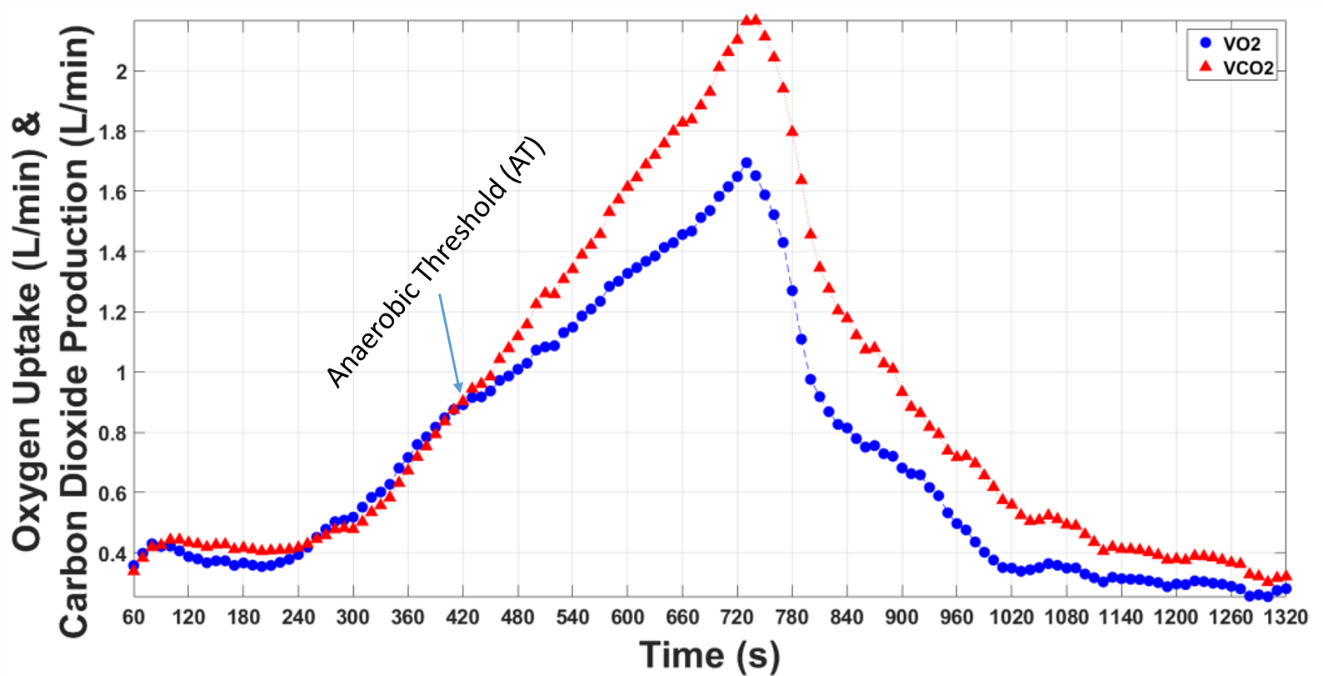 |
| (c) | 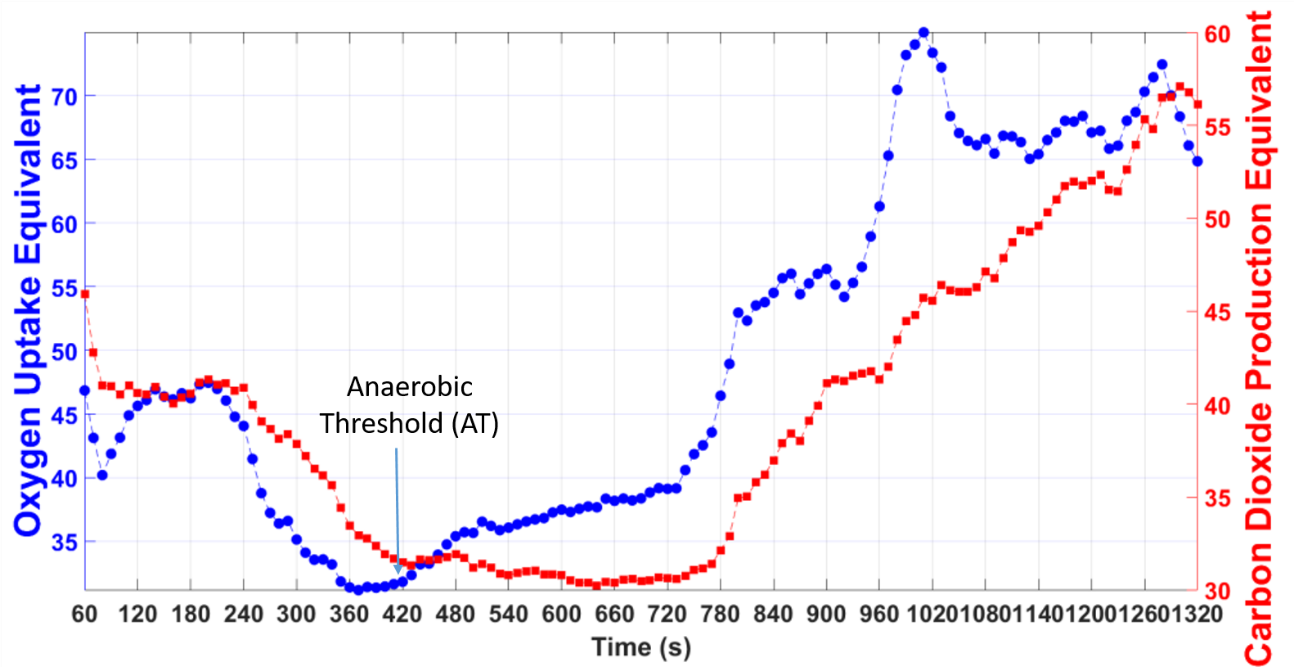 |

**Figure S6**: The determination of ventilatory AT from CPET system data. (a) The V-slope approach plots carbon dioxide against oxygen uptake (blue), the timeline of oxygen uptake (red) was shown; (b) Plots of the metabolic variables - oxygen uptake (blue) and carbon dioxide production (red) over time; (c) The ventilatory equivalents are minute ventilation dived by oxygen uptake (blue) and carbon dioxide production (black) plotted over time. *(In this sample, the AT occurred at 420s, which was exercise stage 2).*

**Figure 1** (main manuscript) illustrates the breakpoint of sound intensity and the related nadir of ventilatory equivalents (V_E_/ VO_2_, V_E_/ VCO_2_ ), which are parameters used for ventilatory AT determination using ventilatory method[^4^](#_ENREF_4). Based on these observations, we hypothesized that the breakpoints in the sound related features were associated with ventilatory AT. Therefore, two independent inspectors (CF and SK) annotated the breakpoints in all the features. Inspectors were blinded to the reference CPET data during the annotation of acoustic signals. For more clear demonstration of the breakpoints the cumulative mean (**Eq. S2**) and variance (**Eq. S3**) of the features were used[^19^](#_ENREF_19). A 'successful detection' was defined as the ability to visually identify a distinct breakpoint in the feature curve. A 'failed detection' occurred only when breathing pattern irregularities or artifacts obscured the signal morphology such that no breakpoint could be identified. The criterion for agreement between evaluators was a difference of *≤20* sec between determinations; discrepancies exceeding this threshold triggered a joint re-assessment to reach consensus, but were not classified as detection failures unless no agreement could be reached.

$cumulative mean{: \mu}_{t}={\sum_{i=1}^{t} {RR}_{i}}/t, t=1,2,\ldots,N$ **Equation S2**

$cumulative variance: {\sigma_{t}}^{2}={\sum_{i=1}^{N} {{RR}_{i}-\mu}_{t}}/t, t=1,2,\ldots,N$ **Equation S3**
